# From Abiotic Stress to Emerging Environmental Pollutants: Expanding Roles of Melatonin and NO in Plant Defense

**DOI:** 10.3390/ijms27125226

**Published:** 2026-06-09

**Authors:** Hanna Witoszek, Anna Wdowikowska, Małgorzata Reda

**Affiliations:** Department of Plant Molecular Physiology, Faculty of Biological Sciences, University of Wrocław, Kanonia 6/8, 50-328 Wrocław, Poland; hanna.witoszek@uwr.edu.pl (H.W.); anna.wdowikowska@uwr.edu.pl (A.W.)

**Keywords:** abiotic stress, greywater, melatonin, microplastics, nanoparticles, nitric oxide, surfactant

## Abstract

In the current era of industrial expansion, the environmental landscape is characterized by an evolving spectrum of contaminants, exposing plants to new types of stress. Plants are forced to employ defensive strategies to survive these conditions. Melatonin (MT) is an amine signaling molecule involved in plant defense processes. Its protective properties in response to various environmental stressors have recently been intensively studied. Melatonin-mediated growth and defense responses result from a multifaceted signaling architecture that involves a wide range of molecules. Among them, nitric oxide (NO) is a key contributor. This review synthesizes existing knowledge of the MT contributions in mitigating well-established abiotic stresses, particularly in connection with NO signaling, and explores the potential to apply these findings to emerging environmental pollutants, such as microplastics, nanoparticles, and surfactants.

## 1. Introduction

Melatonin (N-acetyl-5-methoxytryptamine, MT) is a biomolecule commonly known as an animal hormone that regulates circadian rhythms. It was discovered in the pineal gland of cattle in 1958 [1]. However, it did not attract much interest among plant researchers until 1995, when it was confirmed in Indian spinach, cucumber, cabbage, carrot, strawberry, and many other plants [2,3]. Since then, research on phytomelatonin (phyto-MT) has been developing, and it has become even more dynamic in recent years. Melatonin plays a role in several aspects of plant function, including seed germination [4], flowering [5], and root regeneration [6]. These diverse functions are attributed to the amphiphilic nature of MT [7]. However, the most interesting role of MT is its ability to regulate plant responses to abiotic stress, particularly by managing oxidative damage and modulating reactive oxygen species (ROS) and reactive nitrogen species (RNS) levels [8]. Melatonin has also been shown to increase the levels of defense compounds such as reduced glutathione (GSH) [9]. Nitric oxide (NO), in turn, emerges as a key intermediary molecule in the interaction between melatonin and ROS and RNS [10]. Nitric oxide is a free radical signaling molecule that plays multiple roles in regulating many processes in plants throughout their life span at both physiological and developmental levels [11]. In the plant kingdom, NO was first identified in soybean [12]. Endogenous NO is derived from L-arginine and/or nitrites. However, the specific pathways and their importance in determining the final NO levels in cells are controversial in plant biology, as many possible synthesis pathways have been proposed in plants [13]. The best described one is the synthesis of NO through the reduction of nitrite by nitrate reductase (NR) [14]. Melatonin and NO are interconnected in many ways. Melatonin can modulate NO levels by regulating NR and *S*-nitrosoglutathione reductase (GSNOR) expression [15]. Moreover, they can interact with each other, most notably by the formation of *N*-nitrosomelatonin (NOMela) [16]. Their signaling pathways intersect to maintain redox homeostasis, activate mitogen-activated protein kinase (MAPK) cascades, and regulate ion levels [17]. The mechanisms of those interactions have also been studied because of their importance in alleviating various abiotic stresses [18]. Although many studies have acknowledged that MT can be a powerful factor in maintaining plant growth and functioning under stress conditions, there is a shortage of research regarding emerging pollutants, such as micro/nanoplastics, surfactants in greywater, or nanoparticles. These pollutants pose a threat to plants not only in agriculture but also in many other areas worldwide, as they can be found in increasingly higher concentrations in soil and water [19,20,21]. In an ever-changing environment, it is important to consider MT as a potential approach to mitigate the effects of anthropogenic environmental pollutants, as it plays an established role in mitigating “classic” stressors. When applied exogenously to plants, MT can enhance plant productivity and yield and improve the nutritional value of edible plant products [22]. The objective of this review is to discuss recent studies that explore the features of MT and NO crosstalk in mitigating abiotic stress and evaluate the potential of these molecules in improving plants’ defense systems against new stressors that arise with changing environmental conditions.

## 2. Melatonin and NO—Two Players in One Game

### 2.1. Melatonin and Nitric Oxide Biosynthesis Pathways

The biosynthesis pathway of phyto-MT in plant cells is mostly compartmentalized within the chloroplasts and mitochondria, with the synthesis of some intermediates occurring in the cytoplasm [23,24,25]. The process is initiated by the precursor amino acid tryptophan, the same as in animals. However, unlike animals, which must acquire tryptophan through dietary intake, plants can synthesize this amino acid de novo via the shikimic acid pathway [26,27].

In most plant species studied to date, tryptophan is decarboxylated into tryptamine by tryptophan decarboxylase (TDC) [28,29,30]. In the next step of phyto-MT biosynthesis, tryptamine 5-hydroxylase (T5H) mediates hydroxylation of tryptamine to 5-hydroxytryptamine (serotonin) [31]. Notably, the plant pathway diverges from the classic animal model. Although animals primarily use tryptophan hydroxylase (TPH) to form 5-hydroxytryptophan before converting it into 5-hydroxytryptamine, this specific intermediate is less prevalent in plants. This has been confirmed in select plant species such as *Griffonia simplicifolia* and *Elymus repens* [32]. The final stages of the pathway involve a coordinated conversion of serotonin into melatonin via two potential routes. Serotonin is either acetylated by serotonin *N*-acetyltransferase (SNAT) to form *N*-acetylserotonin or methylated by *N*-acetylserotonin methyltransferase (ASMT) or caffeic acid *O*-methyltransferase (COMT) to produce 5-methoxytryptamine [33]. These intermediates are converted into the final melatonin molecule by the reciprocal actions of ASMT/COMT and SNAT, respectively. For decades, the lack of a confirmed plant receptor has cast doubt on the classification of phyto-MT as a phytohormone. This changed when the melatonin receptor (CAND2/PMTR1) was identified in *Arabidopsis*, where it was observed binding to MT and interacting with the G-protein α subunit 1 (GPA1) [34,35]. Evidence of its receptor function has been confirmed by research, demonstrating its key role in melatonin-mediated plant responses, including diurnal stomatal closure [36] and tolerance to osmotic stress [37] and low-light stress [38].

Melatonin functions as a pleiotropic phytohormone and shows significant structural and functional similarity to indole-3-acetic acid (IAA), with both molecules using tryptophan as their main metabolic precursor and their involvement being in coordination with various stages of plant growth and vegetative development [39,40,41]. Besides auxin, MT has been reported to also interact with other phytohormones, including brassinosteroids [42], jasmonic acid [43,44], salicylic acid [45], ethylene [46], gibberelins [47], and cytokinins [48]. However, among these molecular partnerships, the most significant is that between MT and nitric oxide, the gaseous signaling molecule.

The recognition of NO as a gaseous signaling molecule dates to the 1980s, with significant contributions from Furchgott, Ignarro, and Mura. For their research in this field, they were awarded the Nobel Prize in Physiology or Medicine in 1998 [49]. Nitric oxide can freely penetrate cell membranes via diffusion, owing to its lipophilic character and small size. The half-life of NO is relatively short, and, in most circumstances, it does not exceed 2 s [50]. Nevertheless, during this time NO rapidly reacts with many cellular targets. Similar to melatonin, NO is produced in mitochondria and chloroplasts [51,52]. In plants, NO biosynthesis is generally categorized into (1) enzymatic and (2) non-enzymatic pathways or (1) oxidative/arginine-dependent and (2) reductive/nitrite-dependent pathways. The best-characterized pathway of NO synthesis in plants involves nitrate reductase (NR), a homodimer enzyme localized in the cytosol of plant cells that catalyzes the reduction of nitrate (NO_3_^−^) to nitrite (NO_2_^−^). This is followed by reduction of nitrite to NO by the same NR, xanthine oxidoreductase (XOR) in peroxisomes [53], cytochrome c oxidase (COX) in mitochondria [54] or the plasma membrane-bound form of nitrate reductase (PM-NR) that cooperates with another plasma membrane protein, nitrite:NO reductase (NiNOR) identified in tobacco roots [55,56,57]. Additionally, research into *Chlamydomonas* proposes a pathway including another molybdoenzyme, NO-forming nitrite reductase (NOFNiR) [58]. NOFNiR can reduce nitrites to NO, while NR supplies electrons for this process from NADH [59]. However, the collaborative mechanism of NR:NOFNiR has not been confirmed in higher plants to date [60]. Notably, NO can also be produced non-enzymatically from NO_2_^−^ at acidic pH in the apoplast [61]. Besides the reductive pathways for NO production in higher plants, the existence of an oxidative route, involving NO synthase (NOS), the main producer of NO in animals, bacteria, and fungi [62], remains one of the most debated topics in plant physiology. Although NOS-like activity, sensitive to mammalian NOS inhibitors, has been recorded in plants, the genetic and proteomic identity of supposed plant NOS remains elusive.

### 2.2. Melatonin and Nitric Oxide Interactions

Melatonin and NO represent two molecules essential for plant signaling, with their downstream signaling networks being profoundly interconnected and depending on both synergistic and antagonistic interactions between these molecules. One examples of their pathways crossing is *S*-nitrosylation [63]. *S*-nitrosylation is one of the post-translational modifications induced by NO, and it is defined as a reversible redox-based covalent attachment of a nitroso group to a cysteine (Cys) thiol. This modification is conducted by *S*-nitrosoglutathione reductase (GSNOR) and produces *S*-nitrosothiols (SNOs), which can alter protein conformation, stability, and catalytic activity [64,65]. Melatonin can trigger the accumulation of nitric oxide by downregulation of *S*-nitrosoglutathione reductase (GSNOR) expression [15]. On the other hand, *S*-nitrosylation of MT creates *N*-nitrosomelatonin (NOMela), which emerges as a NO reservoir and donor [16]. Due to the transient nature of NOMela, its properties remain elusive, and further investigation is needed to better understand its localization, formation and breakdown [10].

Melatonin and NO also interact in terms of antioxidative defense. With MT having significant antioxidant properties, it regulates both RNS and ROS levels [66]. It can modulate the transcription levels and activities of several antioxidant enzymes, including superoxide dismutase (SOD), catalase (CAT), peroxidase (POD), ascorbate peroxidase (APX) and glutathione peroxidase (GPX) [67]. Similarly, NO also reacts with ROS [68]. For example, NO can directly bind to O_2_^•−^ and then react with H_2_O_2_, leading to the production of O_2_ and NO_2_, thereby reducing ROS cytotoxicity as a result [68]. However, NO was also found to impair the antioxidant capacity of enzymes by tyrosine nitration, consequently disrupting the cellular redox homeostasis [69,70]. This shows that NO can be considered a protective or toxic molecule, depending on its concentration. At low levels, its role as a signaling molecule is most prominent, but beyond a certain cytotoxic threshold, the accumulation of NO can shift from promoting survival to causing oxidative injury [71]. These dose-dependent NO properties are crucial in demonstrating the dual nature of MT-NO interactions. The application of exogenous melatonin has been shown to trigger either an increase [72,73,74] or a decrease [75,76,77] in NO levels, aiming to maintain NO levels in a physiological range. Therefore, depending on the surrounding physiological context and specific plant species, melatonin can either enhance the NO signaling pathway or act as a NO scavenger. This is crucial, especially with environmental stressors sometimes resulting in high accumulation of NO [76,77].

The physiological effects of NO are largely mediated through its role as a central hub in signaling networks. A primary downstream target of NO is the activation of guanylate cyclase, which triggers the accumulation of cyclic GMP (cGMP) and activates MAPK cascades, specifically MAPK3 and MAPK6. Similarly, NO signaling is associated with calcium (Ca^2+^) flux [78]. Intracellular Ca^2+^ spikes activate calmodulin (CaM), which promotes NO biosynthesis [79]. NO can also modulate Ca^2+^ channels on the plasma membrane, creating a signaling pathway that regulates plant responses to both pathogens and environmental stressors [80]. Similarly, melatonin has also been shown to modulate MAPK cascade and Ca^2+^ contents [81]. Other ions levels (Na^+^, K^+^) are also regulated by melatonin [82], highlighting its role in osmotic adjustment, which enhances plant resistance to stress.

## 3. Melatonin–NO Crosstalk in Abiotic Stress Response

Maintaining homeostasis under environmental pressures is critical for plant productivity. In recent years, the effects of exogenous MT application on plants exposed to various abiotic stressors have been extensively studied (Table 1). MT-NO crosstalk can provide defense mechanisms that integrate various pathways to help the plant in adapting to threats like low temperature, drought, salinity, or heavy metals. The synergistic relationship between MT and NO in mitigating stress responses is discussed below within the response to each of the mentioned factors.

### 3.1. Cold Stress

Low-temperature (LT) stress is a major abiotic constraint that significantly limits crop growth and global productivity. Chilling stress (0–15 °C) can severely disrupt plant physiology, including photosynthesis, respiration, and nutrient uptake [83]. The exogenous application of MT has emerged as a pivotal strategy for enhancing plant cold stress resilience. This protective effect is mediated by a multi-level regulatory framework that depends on different biochemical, physiological, and molecular mechanisms.

In cucumber, melatonin treatment increased the content of Ca^2+^ and calcium-dependent protein kinases (CPKs) and induced the expression of genes encoding Ca^2+^ transporters, the CPK family, and proteins involved in the MAPK cascade and ICE-CBF-COR pathway, which are important components in regulating LT tolerance [81,84]. Additionally, MT modulates endogenous NO homeostasis, though the direction of this regulation is highly context-dependent. For instance, in cucumber, MT treatment suppresses toxic NO accumulation by promoting the binding of the CsZAT10 transcription factor to the *CsGSNOR* promoter, upregulating GSNOR activity [75]. Conversely, in watermelon, MT-induced cold tolerance involves the upregulation of nitrate reductase (ClNR1), which increases NO production to drive downstream CBF activation [72].

Melatonin application can also mitigate cold stress by upregulating the concentrations of mineral elements, improving growth parameters and germination, increasing water and chlorophyll content, and altering antioxidant enzyme activities and mitochondrial respiration, as shown in maize, pepper, rice, soybean, and tomato [38,85,86,87,88,89,90].

Interestingly, it was also reported that MT seed priming can overcome cold stress by polyploidization induced via endoreplication in the basal zone of maize axes [91]. Polyploid cells are naturally more resilient, allowing plants to adapt more effectively to unfavorable environmental changes. Melatonin pretreatment in seeds under chilling conditions has also been shown to restore circadian clock oscillation in barley [92].

It is also worth noting that the protective properties of exogenous MT extend to alleviating injuries from cold stress in fruits subjected to cold storage, such as bananas [93], winter jujubes [94], and plums [95]. Although cold storage is the primary strategy for extending fruit shelf life, low temperatures often trigger chilling injury. Exogenous MT mitigates cold-induced damage by activating ROS scavenging, while simultaneously elevating levels of signaling molecules such as NO and stress-responsive metabolites, including polyamines, proline, and GABA [96,97,98].

### 3.2. Drought Stress

Exogenous melatonin has also been shown to reduce drought stress. Exposure to drought induces systemic oxidative and nitrosative damage resulting from the production of ROS and RNS in stressed plants. Moreover, water scarcity severely impairs vital plant functions, such as photosynthesis and nutrient transport, leading to inhibited growth and, in extreme cases, programmed cell death [99,100].

Maize appears to be one of the most well-researched plants in this context. Several studies have reported improved photosynthetic activity (mainly through regulation of stomatal opening), reduced oxidative damage, improved nitrogen metabolism and increased dry matter production in drought-stressed maize treated with melatonin [101,102,103,104]. In the context of melatonin–NO crosstalk, the most fascinating findings seem to be those related to stomatal opening regulation. It is known that NO participates in the stomata opening/closing mechanism under stress conditions [105,106], possibly by NO-controlled *S*-nitrosylation of MAP kinase MPK6 at Cys-201, inhibiting its kinase activity [107]. Moreover, the effect of MT treatment on nitrogen metabolism also reflects its connection to NO by increasing the activity of NR, along with upregulation of the corresponding transcripts [103]. As mentioned before, NR is the most prominent producer of NO in plants; therefore, its heightened activity, along with elevated antioxidant enzyme activities, as shown in several studies, makes the melatonin–NO crosstalk a prominent player in creating drought resilience in plants. Under PEG-induced drought, exogenous MT exhibited similar alleviating effects in maize, with an additional report of decreased ABA levels by regulating the expression of genes related to the ABA metabolic pathway [108].

A decrease in ABA levels and downregulation of ABA-related gene expression were also major findings of the study on melatonin–NO crosstalk in drought-treated soybean [109]. Increased *gmNR* gene expression, significantly higher antioxidant enzyme activities, and reduced lipid peroxidation affirm the joint role of melatonin and NO in ROS scavenging. Another study proposed that MT may also improve drought tolerance in soybean by activating enzymes related to glucose metabolism and regulating the expression of related genes [110].

Increased antioxidant enzyme activity and improved photosynthesis were also reported in other plants, such as apple, rose, and potato, subjected to drought and treated with MT [111,112,113]. Although similar findings were obtained with alfalfa, reduced NO content and NR activity in plants treated with MT under drought stress were observed compared to plants growing in drought conditions and not treated with MT [114]. This suggests that melatonin may also downregulate NO synthesis in some cases, possibly limiting nitrosative stress.

### 3.3. Heavy Metal Stress

Industrialized areas are increasingly polluted by heavy metals. Plants can accumulate metals from the soil, posing risks to agriculture and food safety. Many heavy metals are essential micronutrients for plant health, as they activate enzymes or act as cofactors, such as copper (Cu), iron (Fe), or zinc (Zn). However, non-essential heavy metals, including aluminum (Al), cadmium (Cd), and lead (Pb), can cause various phytotoxic effects [115]. On the other hand, the accumulation of heavy metals by plants that are resilient to their detrimental effects (hyperaccumulators) can be a form of phytoremediation, leading to the improvement of contaminated soil [116]. Nevertheless, most plants are prone to heavy metal-induced stress, reacting with a complex physiological crisis. Heavy metals mostly hinder primary metabolism and cause oxidative instability. By disrupting the balance between ROS production and scavenging, these metals reduce photosynthetic efficiency and mineral absorption in plants. This leads to reduced growth and atypical morphology [117]. The role of MT-NO crosstalk in mitigating these negative effects has been shown in numerous studies.

#### 3.3.1. Aluminum

Aluminum toxicity initiates a phytotoxic cascade that impairs vegetative development. The critical threshold for Al-induced root damage varies among species. Sensitive plants exhibit significant root growth inhibition at concentrations as low as 5–10 μM, whereas tolerant species demonstrate resilience even at levels exceeding 200 μM, especially *Camellia sinensis*, which is very tolerant to Al toxicity at over 1000 μM [118].

In *Arabidopsis*, Al exposure downregulates the expression of *AtSNAT*, leading to a lower level of endogenous MT, which correlates with an overproduction of NO that arrests the root cell cycle [76]. Notably, exogenous melatonin application reverses this phenotype by suppressing Al-induced NO accumulation and restoring root growth [76,77]. Melatonin has also been shown to mitigate Al stress in rice and soybean by cell wall modifications and induction of Al vacuolar sequestration, along with increased activities of antioxidant enzymes [77,119,120].

Moreover, melatonin treatment decreased Al accumulation in apple roots, with RNA-seq data showing that *MdMAPK3* expression changed under Al and MT treatments, consistent with reports on MAPK involvement in the melatonin signaling pathway [121]. At the molecular level, MT was also found to alleviate Al-induced DNA degradation and excessive endoreduplication in the root apical meristem, thereby preserving QC identity, improving photosynthetic performance and reducing oxidative damage in the medicinal plant *Pfaffia glomerata* [122].

#### 3.3.2. Cadmium

Cd is one of the most dangerous and toxic heavy metals that plants are exposed to in the environment. Therefore, methods to counteract its toxic effects, particularly those related to melatonin and NO, are among the most frequently studied. A direct link between the melatonin–NO relationship and the mitigation of cadmium stress has been observed in the periwinkle [123]. Melatonin increased endogenous NO levels, thereby enhancing plant tolerance to Cd. In contrast, using NO scavenger 2-(4-carboxyphenyl)-4,4,5,5-tetramethylimidazoline-1-oxyl-3-oxide (cPTIO) suppressed the protective effects of MT. The stress-relieving effects of MT and NO have been associated with increased activities of superoxide dismutase (SOD) and ascorbate peroxidase (APX) and reduced H_2_O_2_ and MDA content, indicating lower oxidative stress and lipid peroxidation [123]. A possible way that melatonin interrupts the chain of lipid peroxidation is by reacting with lipid peroxyl (LOO∙) radicals [124].

Moreover, the combined application of MT and NO donor sodium nitroprusside (SNP) was more effective than treatment with either MT or SNP alone in alleviating Cd stress in eggplants [125], highlighting and confirming the importance of their crosstalk in enhancing heavy metal stress resilience in plants.

Similar findings, including restored photosynthesis, improved fresh and dry weights, improved growth and stomatal conductance, lowered root Cd uptake, reduced Cd accumulation, alleviated H_2_O_2_ and MDA production, increased antioxidant enzyme (SOD, CAT, POD, APX) activities and increased expression of genes encoding proteins involved in these processes, have also been shown in apple [126], orchid [127], cotton [67], cucumber [128], maize [129], mallow [130], tobacco [131], strawberry [132], and wheat [133].

Melatonin has also been linked to the mitigation of Cd stress by reprogramming sulfur metabolism and glutathione *S*-transferase (GST) activity [134]. GST is another enzyme that can interact with NO in cellular signaling [135]. MT also affects nitrogen assimilation in basket willows, increasing glutamine content under Cd stress [136]. Glutamine is a precursor of arginine, which can participate in NO biosynthesis [137]. Notably, there have been instances where exogenous MT treatment has had detrimental effects on jujube seedlings, inhibiting growth and reducing biomass, SOD activity, and photosynthetic pigments [138]. However, it is worth acknowledging that suitable MT concentrations for exogenous treatment vary widely across species (see Table 1), and in different studies, jujube fruits have responded positively to melatonin treatment, albeit post-harvest [139,140].

#### 3.3.3. Other Heavy Metals

Stress mitigation via melatonin–NO crosstalk has also been shown to mitigate stress caused by other metals. Melatonin treatment in cotton plants subjected to chromium (Cr) stress improved height and biomass, as well as chlorophyll content and photosynthetic parameters. However, adding NO scavenger cPTIO restricted these beneficial results, confirming the involvement of MT-NO interactions in the mitigation of the harmful effects of metals on plants [141]. A similar method was used in a study on lead (Pb) stress in maize, in which cPTIO abolished improvements in the antioxidant defense systems and MT-induced resilience [73]. Indirect connection to NO can also be made based on results from a study on melatonin treatment of rosemary under arsenic (As) stress, with improved SOD, CAT, POD, and APX activities and MT’s protection of chloroplasts from injuries caused by excess ROS [142].

### 3.4. Salt Stress

Salt stress induces multifaceted damage in plants, primarily driven by the excessive accumulation of sodium (Na^+^) ions in plant tissues. Salt stress affects plants in two main ways: (I) causing early osmotic stress and (II) late ionic toxicity [143]. In the case of early osmotic stress, external osmotic imbalance causes impaired water uptake and cell turgor [144], whereas late ionic toxicity is related to absorption and accumulation of excessive amounts of sodium and chloride ions and simultaneous loss of potassium (K^+^) ions [145], It leads to disruption of cellular homeostasis, enzyme activity, photosynthesis, and antioxidant processes and finally to cell damage or death. The protective properties of MT treatment can help prevent these negative repercussions of salt stress by altering the physiological, morphological, and biochemical responses of plants [146]. At the molecular level, melatonin can also upregulate the expression of genes involved in maintaining salt tolerance, such as components of the Salt Overly Sensitive (SOS) pathway, including *SOS1* (plasma membrane Na^+^/H^+^ antiporter) [147].

A recent study on the effects of melatonin on alfalfa grown hydroponically under increased salinity conditions showed that foliar spraying of MT restored ionic and redox homeostasis by reducing Na^+^ accumulation and enhancing ROS scavenging and antioxidant enzyme activity in plant tissues. Increased production of proline, soluble proteins, and soluble sugars has also been observed, resulting in the adjustment of osmotic homeostasis [148]. Moreover, transcriptomic analysis (RNA-seq) revealed that during the initial stage of stress, MT induces an “energy saving” strategy by significantly inhibiting ribosome biogenesis and related transcriptional processes, while simultaneously prioritizing the mobilization of antioxidant enzymatic systems (SOD, POD, and CAT) and maintaining the integrity of the photosynthetic apparatus. However, as stress persists, there is a shift towards the activation of the ABA signaling network and lignin metabolic pathways to maintain membrane integrity and cellular osmotic balance [149]. In addition to other phytohormones (gibberellins, IAA), melatonin modulated ABA levels in salt-stressed soybean and *Sophora alopecuroides* [150,151]. The melatonin–NO relationship was reported to regulate salt tolerance in tomatoes, where the beneficial role of melatonin application was confirmed in improving plant growth, biomass, and chlorophyll and proline metabolism, strengthening the antioxidant defense system, and maintaining ion balance by modulating the gene expression of NHX transporters involved in Na^+^/K^+^ homeostasis. All these effects were inhibited by cPTIO, establishing that tomato seedling tolerance to salt stress is dependent on melatonin–NO interactions [74].

It has also been hypothesized that melatonin may alleviate salt stress by modulating the polyamine metabolic pathway. In a study on wheat, exogenous melatonin increased the conversion of polyamine from biosynthetic precursors (arginine and methionine) and reduced salt-induced polyamine degradation [152]. Similarly, combined treatment with MT and putrescine (Put) significantly improved the physiology of green beans under salt stress by optimizing the antioxidant defense system and simultaneously reducing the activities of diamine oxidase (DAO) and polyamine oxidase (PAO) [153]. The polyamine and NO signaling pathways are known to intersect, as polyamines can induce NO biosynthesis, which is a downstream component of melatonin signaling [154].

In various plants such as radish [155], rice [156] turfgrass [157], soybean [151], thyme [158], tomato [159] and watermelon [160], exogenous melatonin has been found to alleviate salinity stress through several physiological improvements, including reduced growth inhibition, increased RWC and photosynthetic pigment levels (chlorophylls and carotenoids), enhanced antioxidant activity and osmotic adjustment, and reduced membrane lipid peroxidation. Improvements in morphological parameters (biomass, leaf number and area, plant height, and root length), as well as photosynthetic and antioxidant parameters, have also been confirmed in maize [161,162,163]. Although the involvement of NO was not directly confirmed in these studies, it can be theorized based on the established role of NO in mitigating these stress responses.

**Table 1:** Effects of exogenous MT treatment in selected abiotic stresses (bold text highlights co-evaluation of the NO responses).

Type of Stress		Plant	MT Concentration	MT Application Technique	Effects of Melatonin Treatment	References
Cold		*Capsicum annuum* (pepper)	200 μM	Foliar spray	Root growth **↑**, ROS (H_2_O_2_, O_2_^−^) ↓, MDA ↓, antioxidant enzymes (SOD, CAT, POD, APX) ↑, AsA-GSH cycle ↑, soluble sugars ↑, soluble proteins ↑, osmotic regulation ↑, membrane stability ↑	[38]
		*Citrullus lanatus* (watermelon)	150 μM	Foliar spray/genetic overexpression	CBF pathway activation ↑, gene expression (*ClCBF1*, *ClCOR47*, *ClNR1*, *ClLCD*) ↑, **NO levels** ↑, H_2_S ↑, **signaling crosstalk (NO–H_2_S loop)** ↑, MDA ↓, photosystem efficiency ↑	[72]
		*Cucumis sativus* (cucumber)	100 μM	Foliar spray	Growth ↑, ROS ↓, MDA ↓, lipid peroxidation ↓, **NO accumulation ↓**, **GSNOR activity and expression** ↑, gene expression (*CsZAT10*, *CNGC*, *CPKs*, *ICE-CBF-COR*, *MAPK*) ↑, **NO signaling regulation** ↑, photosynthesis ↑, antioxidant defense ↑, Ca^2+^ content ↑, CPKs signaling ↑	[75,81]
		*Glycine max* (soybean)	5 μM	Foliar spray	Growth ↑, mineral nutrients (K, Ca, Mg) ↑, antioxidant gene expression ↑, antioxidant enzymes ↑, ROS ↓, oxidative damage ↓	[88]
		*Oryza sativa* (rice)	20–100 μM	Seed priming + added to the nutrient solution + foliar spray	Growth inhibition ↓, photosynthesis (PSII activity, chlorophyll fluorescence) ↑, ROS ↓, MDA ↓, cell death ↓, antioxidant enzymes (SOD, POD, CAT) ↑, non-enzymatic antioxidants ↑	[87]
		*Solanum lycopersicum* (tomato)	100 μM	Foliar spray	Photosynthesis (PSII efficiency) ↑, antioxidant capacity ↑, GSH/GSSG and AsA/DHA ratios ↑, ROS ↓, lipid peroxidation ↓, redox homeostasis ↑	[89]
		*Zea mays* (maize)	100, 1000 μM	Foliar spray	Growth (root length, plant height, leaf area) ↑, chlorophyll ↑, mitochondrial respiration ↑, ATP production ↑, TCA cycle enzymes ↑, antioxidant enzymes (SOD, CAT, APX) ↑, ROS (H_2_O_2_, O_2_^−^) ↓, MDA ↓, electrolyte leakage ↓, oxidative damage ↓, mineral elements ↑, relative water content ↑, membrane stability ↑	[85,86]
			50 μM	Seed priming	Germination rate and potential ↑, embryo growth ↑, antioxidant enzymes (SOD, POD, CAT, APX, GSH-PX, GST) ↑, ROS (H_2_O_2_) ↓, MDA ↓, lipid peroxidation ↓, protein oxidation ↓, endoreplication ↑, soluble proteins ↑, soluble sugars ↑, starch metabolism ↑	[90,91]
Drought		*Glycine max* (soybean)	100 μM	Foliar spray	Dry matter accumulation ↑, glucose metabolism ↑, sucrose and starch metabolism ↑, metabolized sugars ↑, MDA ↓, yield ↑	[110]
				Foliar spray	Growth (biomass) ↑, photosynthesis ↑, water content ↑, ROS ↓, lipid peroxidation ↓, antioxidant enzymes ↑, **NO signaling** ↑, ABA ↓, transcription factors (*WRKY*, *MYB*) ↑	[109]
		*Malus domestica* (apple)	100 μM	Added to soil	Leaf senescence ↓, chlorophyll degradation ↓, photosynthesis (PSII efficiency, gas exchange) ↑, ROS (H_2_O_2_) ↓, antioxidant enzymes (CAT, POD, APX, AsA–GSH cycle) ↑, oxidative stress ↓	[111]
		*Medicago sativa* (alfalfa)	10 μM	Added to the nutrient solution	Chlorophyll fluorescence ↑, stomatal conductance ↑, ROS (H_2_O_2_) ↓, **NO levels** ↓, MDA ↓, antioxidant enzymes (SOD, CAT, APX) ↑, nitro-oxidative homeostasis ↑, proline metabolism ↑	[114]
		*Rosa centifolia* (rose)	100 μM	Foliar spray	Growth (height, flower yield, biomass) ↑, photosynthesis efficiency ↑, chlorophyll, carotenoids ↑, antioxidant enzymes (SOD, CAT) ↑, proline, glycine betaine, soluble proteins ↑, ROS (H_2_O_2_) ↓, MDA ↓, oxidative stress ↓	[112]
		*Solanum tuberosum* (potato)	50–150 μM	Foliar spray	Growth ↑, photosynthetic efficiency ↑, ROS ↓, MDA ↓, cell death ↓, antioxidant enzymes (SOD, CAT, POD) ↑, osmolytes (proline, soluble sugars) ↑, gene expression (*MYB*, *NAC*, *ERF*) ↑, hormone signaling ↑, carbon metabolism ↑	[113]
		*Zea mays* (maize)	100 μM	Foliar spray	Photosynthetic capacity (CO_2_ assimilation, enzyme activity, chlorophyll) ↑, dry matter accumulation ↑, relative water content ↑, antioxidant enzymes (SOD, CAT, POD, AsA–GSH cycle) ↑, ROS (H_2_O_2_) ↓, MDA ↓, oxidative damage ↓, stomatal reopening ↑, ABA accumulation ↓, carbon metabolism (sucrose, starch synthesis) ↑, nitrogen metabolism ↑, proline ↑,	[101,103,108]
			20–100 μM	Foliar spray + added to the irrigation water	Photosynthesis (PSII activity, photochemical efficiency) ↑, chlorophyll ↑, ROS ↓, cell death ↓, lipid peroxidation ↓, antioxidant system ↑	[104]
			1000 μM	Added to the nutrient solution	PSII efficiency ↑, photoprotection ↑, antioxidant role ↑, interaction with phytohormones (ABA, SA, JA) ↑, stress memory/recovery ↑	[102]
Heavy metals	Al	*Arabidopsis thaliana*	10 μM	Added to medium	Root growth ↑, **NO production** ↓, cell cycle activity ↑	[76]
		*Glycine max* (soybean)	0.1–200 μM	Added to the nutrient solution	0.1–1 μM: root growth ↑, H_2_O_2_ ↓, SOD, POD, CAT ↑ 100–200 μM: root growth ↓, antioxidant enzymes ↓, Al accumulation in cell wall ↓, pectin, hemicellulose ↓, cell wall rigidity ↓, lignin synthesis ↓, Al sequestration into vacuole ↑, expression of Al transport genes (*ALS1*, *Nrat1*, *CDT3*, *IREG3*) modulated	[119,120]
		*Oryza sativa* (rice)	20 μM	Added to the nutrient solution	Al accumulation ↓ (root tips), *OsSTAR1*, *OsSTAR2*, *OsNRAT1* expression ↓, *OsALS1* ↑, hemicellulose content ↑, Al sequestration into vacuole ↑, **NO accumulation** ↓, Al resistance ↑	[77]
		*Pfaffia glomerata*	100 μM	Added to medium	Photosynthetic pigments ↑, photosynthetic rate ↑, carbohydrates, amino acids, proteins ↑, CAT, SOD, GABA ↑, DNA damage ↓, root growth ↑, Al accumulation ↓, Al adherence to roots ↓, antioxidant defense ↑	[122]
		*Catharanthus roseus* (periwinkle)	100 μM	Added to the nutrient solution	Biomass ↑, chlorophyll ↑, relative water content ↑, proline ↑, antioxidant enzymes (SOD, APX) ↑, H_2_O_2_ ↓, MDA ↓, alkaloid (vinblastine, vincristine) biosynthesis ↑, gene expression (*D4H*, *DAT*) ↑, **NO signaling ↑**	[123]
	Cd	*Cucumis sativus* (cucumber)	150 μM	Foliar spray	Leaf area ↑, chlorophyll ↑, photosynthetic rate ↑, stomatal conductance ↑, transpiration ↑, PSII efficiency ↑, Cd accumulation ↓ (roots)	[128]
		*Fragaria × ananasa* (strawberry)	10–200 μM	Foliar spray	Growth (biomass, root/shoot length) ↑, chlorophyll ↑, antioxidant enzymes (SOD, POD, CAT, APX) ↑, soluble protein ↑, anthocyanins ↑, ROS ↓, MDA ↓, senescence ↓	[132]
		*Gossypium hirsutum* (cotton)	50 μM	Foliar spray	Cd accumulation ↓, ROS (H_2_O_2_, O_2_^−^) ↓, MDA ↓, antioxidant enzymes (SOD, CAT, POD, APX, GST) ↑, cell wall components (cellulose, hemicellulose, pectin) ↑, Cd binding capacity ↑, Cd transporter genes ↓, Cd sequestration ↑	[67]
		*Malus* spp. (apple)	100 μM	Added to the nutrient solution	Growth (biomass, photosynthesis) ↑, photosynthetic pigments ↑, ROS ↓, MDA ↓, antioxidants ↑, Cd uptake ↓ (roots), Cd accumulation ↓ (leaves), Cd translocation ↓, expression of Cd transport/detox genes (*HA7*, *NRAMPs*, *HMA4*, *ABCC1*) modulated, Cd detoxification ↑	[126]
		*Nicotiana tabacum* (tobacco)	25–250 μM	Foliar spray	Growth inhibition ↓, photosynthesis ↑, ROS (H_2_O_2_, O_2_^−^) ↓, antioxidant enzymes (SOD, CAT, APX) ↑, Cd accumulation ↓, Cd sequestration (cell wall, vacuole) ↑, expression of Cd transport genes (*IRT1*, *HMA2/4 ↓*; *HMA3* ↑)	[131]
		*Solanum melongena* (eggplant)	100 μM	Foliar spray	Growth (biomass, chlorophyll, protein, water status) ↑, antioxidant enzymes (SOD, CAT, POD) ↑, ROS (H_2_O_2_) ↓, MDA ↓, electrolyte leakage ↓, Cd uptake ↓, Cd translocation ↓	[125]
		*Triticum aestivum* (wheat)	0.1–1000 μM	Added to the nutrient solution	Growth (plant height, biomass, root length) ↑, H_2_O_2_ ↓ (homeostasis restored), GSH ↑, GSH/GSSG ratio ↑, antioxidant enzymes (SOD, APX) ↑, root oxidative detox capacity ↑	[133]
	Cd	*Zea mays* (maize)	200 μM	Foliar spray	Growth (biomass, seedling growth) ↑, antioxidant enzymes (SOD, POD, CAT) ↑, nitrogen metabolism enzymes ↑, Cd accumulation ↑, Cd uptake ↑	[129]
		*Zizyphus acidojujuba*	50–200 μM	Added to the nutrient solution	Growth ↓, biomass ↓, photosynthetic pigments ↓, antioxidant enzymes (SOD, POD) ↓, soluble protein ↓, Cd accumulation ↑, Cd translocation ↑	[138]
	Cr	*Gossypium hirsutum* (cotton)	100 μM	Added to the nutrient solution	Growth (height, biomass) ↑, photosynthetic pigments ↑, antioxidant enzymes (SOD, APX, CAT, POD) ↑, ROS (H_2_O_2_) ↓, MDA ↓, Cr accumulation ↓, GSH ↑, phytochelatins ↑, cysteine metabolism ↑, gene expression (*CYC*, *CAS*, *DES*, *SSCS*) ↑, Cr sequestration into vacuole ↑	[141]
	Pb	*Zea mays* (maize)	50–100 μM	Foliar spray	Growth (biomass) ↑, chlorophyll ↑, PSII efficiency ↑, K^+^, Ca^2+^ ↑, ROS (H_2_O_2_) ↓, MDA ↓, electrolyte leakage ↓, proline ↓, Pb accumulation ↓, **NO levels** ↑, antioxidant enzymes ↑	[73]
	As	*Rosmarinus officinalis* (rosemary)	25–50 μM	Foliar spray	Growth (biomass) ↑, photosynthetic pigments ↑, ion balance ↑, relative water content ↑, osmolytes ↑, antioxidant enzymes (SOD, CAT, POD, APX) ↑, ROS ↓, membrane stability ↑, phenols, flavonoids, anthocyanins ↑, essential oil yield ↑, as accumulation ↓	[142]
Salt		*Citrullus lanatus* (watermelon)	50–500 μM	Added to the irrigation water	Photosynthesis ↑, stomatal conductance ↑, light energy absorption ↑, electron transport (PSII) ↑, ROS ↓, antioxidant enzymes ↑, redox homeostasis ↑, membrane damage ↓, oxidative stress ↓	[160]
		*Festuca elata* (turfgrass)	50–150 μM	Foliar spray	Growth (plant height, leaf area) ↑, relative water content ↑, chlorophyll, carotenoids ↑, osmolytes (proline, soluble protein) ↑, antioxidant enzymes (SOD, POD) ↑, ROS ↓, MDA ↓, membrane protection ↑	[157]
		*Glycine max* (soybean)	50–250 μM	Seed priming + added to the nutrient solution	Germination ↑, growth ↑, antioxidant enzymes (SOD, POD, CAT) ↑, ROS (H_2_O_2_) ↓, MDA ↓, osmolytes ↑, ABA ↓, hormones (IAA, GA, JA) ↑, hormonal homeostasis ↑, stress-to-growth transition ↑	[151]
		*Medicago sativa* (alfalfa)	50–150 μM	Foliar spray	Growth (plant height, leaf area, root morphology) ↑, photosynthesis ↑, MDA ↓, ROS ↓, antioxidant enzymes (SOD, POD, CAT) ↑, soluble sugars ↑, osmoprotection ↑, gene expression (ABA signaling) ↑, energy metabolism (ribosome-related genes ↓), secondary metabolism ↑, Na^+^ ↓, K^+^ ↑, ion homeostasis ↑, osmolytes (proline, soluble sugars, proteins) ↑, antioxidant enzymes ↑, membrane stability ↑	[148,149]
		*Momordica charantia* (bitter melon)	75–150 μM	Added to the nutrient solution	Photosynthesis ↑, relative water content ↑, K^+^, Ca^2+^, P ↑, Na^+^, Cl^−^ ↓, ion homeostasis ↑, ROS (H_2_O_2_) ↓, MDA ↓, antioxidant system ↑, gene expression (*WRKY1*, *SOS1*) ↑	[147]
		*Oryza sativa* (rice)	50–200 μM	Foliar spray	Photosynthesis ↑, sucrose, starch ↑, relative water content ↑, ROS ↓, antioxidant capacity ↑, xanthophyll cycle ↑, photoprotection ↑, photosynthetic enzymes ↑, energy dissipation balance ↑	[156]
Salt		*Phaseolus vulgaris* (snap bean)	20 μM	Added to the nutrient solution	Growth (fresh/dry weight) ↑, chlorophyll (a, b), carotenoids ↑, relative water content ↑, osmolytes (proline, soluble sugars) ↑, K^+^, Ca ↑, Na^+^ ↓, Na/K ratio ↓, ion homeostasis ↑, antioxidant enzymes (SOD, CAT, APX, POX) ↑, ROS (H_2_O_2_) ↓, MDA ↓, membrane stability ↑, polyamine catabolism ↓	[153]
		*Raphanus sativus* (radish)	50–100 μM	Added to the irrigation water	Photosynthesis (CO_2_ assimilation, stomatal conductance) ↑, chlorophyll ↑, relative water content ↑, membrane stability ↑, Na^+^, Cl^−^ ↓, nutrient balance ↑, ion homeostasis ↑, physiological performance ↑, salt tolerance ↑	[155]
		*Solanum lycopersicum* (tomato)	150 μM	Added to the nutrient solution	Plant height ↑, biomass ↑, photosynthesis ↑, chlorophyll ↑, proline metabolism ↑, nitrogen metabolism ↑, antioxidant enzymes ↑, ROS (O_2_^−^, H_2_O_2_) ↓, MDA ↓, electrolyte leakage ↓, **NO production ↑,** *NHX* gene expression ↑, AsA–GSH cycle ↑, Na^+^ ↓, K^+^/Na^+^ homeostasis ↑, nutrient accumulation ↑, membrane stability ↑	[74,159]
		*Sophora alopecuroides*	50–250 μM	Seed priming	Germination ↑, biomass ↑, photosynthesis ↑, stomatal conductance ↑, ROS ↓, MDA ↓, antioxidant system (SOD, CAT, POD, flavonoids, carotenoids) ↑, hormones (IAA, GA) ↑, proline ↑, Na^+^/K^+^ balance ↑, transcriptomic changes ↑	[150]
		*Thymus daenensis* (thyme)	100 μM	Foliar spray	Biomass (fresh, dry weight) ↑, chlorophyll (a, b, total), carotenoids ↑, photosystem II efficiency ↑, electron transport ↑, energy dissipation ↓, essential oil content ↑, photosynthetic performance ↑	[158]
		*Triticum aestivum* (wheat)	1 μM	Added to the nutrient solution	Growth ↑, shoot dry weight ↑, photosynthesis ↑, chlorophyll ↑, PSII efficiency ↑, ROS (H_2_O_2_) ↓, endogenous melatonin ↑, polyamines (putrescine, spermidine, spermine) ↑, polyamine metabolism ↑, IAA ↑, oxidative damage ↓	[152]

↑—increase, ↓—decrease. ABA: abscisic acid; AsA: ascorbic acid; APX: ascorbate peroxidase; CAT: catalase; DHA: dehydroascorbate; GA: gibberellic acid; GABA: gamma-aminobutyric acid; GSH: reduced glutathione; H_2_O_2_: hydrogen peroxide; IAA: indole-3-acetic acid; JA: jasmonic acid; MDA: malondialdehyde; NO: nitric oxide; POD: peroxidase; PSII: photosystem II; ROS: reactive oxygen species; SA: salicylic acid; SOD: superoxide dismutase; TCA: tricarboxylic acid cycle.

## 4. Translating Classic Stress Tolerance Mechanisms: The Prospective Role of Melatonin and Nitric Oxide Against Emerging Pollutants

The worldwide growth of industry and plastic consumption has led to the emergence of a new class of environmental stressors that are now widespread in agricultural soils and irrigation water [164,165,166].

**Figure 1:**
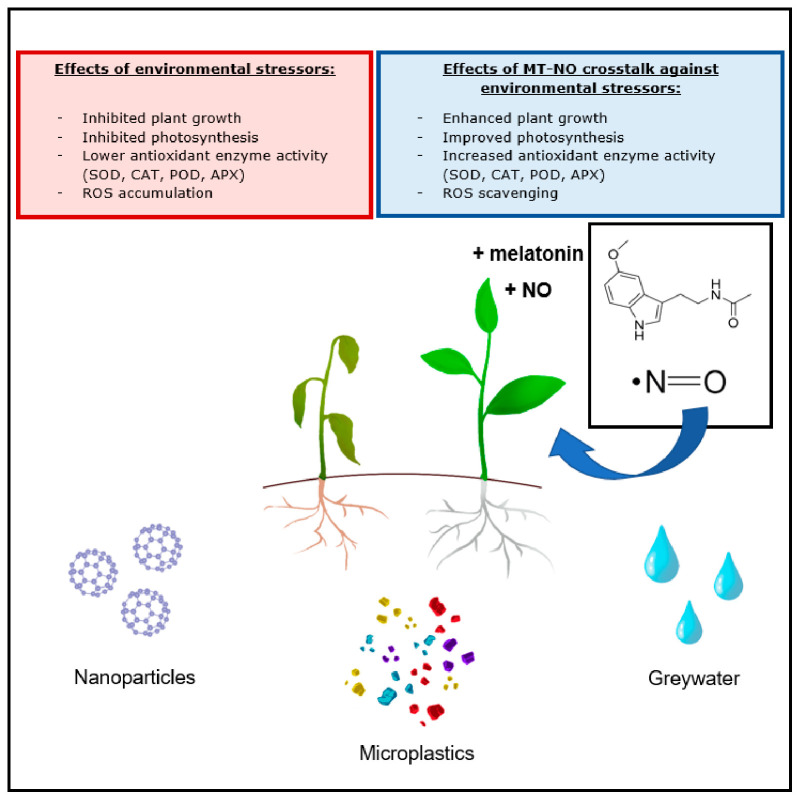
Schematic presentation of MT-NO crosstalk’s protective effects on plant physiology in response to emerging environmental stressors. APX: ascorbate peroxidase; CAT: catalase; NO: nitric oxide; POD: peroxidase; ROS: reactive oxygen species; SOD: superoxide dismutase.

Agricultural ecosystems are currently undergoing rapid transformation, which is defined by novel chemical challenges. The widespread accumulation of microplastics, nanomaterials, and surfactants represents a shift from naturally occurring stressors to synthetic, human-induced ones. Because these “new” pollutants often trigger unpredictable physiological problems in plants, the protective synergy between melatonin and nitric oxide offers a promising strategy to enhance plant resilience in an increasingly contaminated world. These interactions are illustrated in Figure 1.

### 4.1. Microplastics and Nanoplastics

“Microplastics” is a term that describes plastic materials measuring less than 5 mm [167]. Their derivatives, nanoplastics, measure less than 100 nm [168]. Both micro- and nanoplastics are especially alarming novel environmental pollutants, as plastic is a highly resistant material that takes many years to decompose. Microplastics are transported globally through various media, which contributes to their widespread presence in aquatic and terrestrial ecosystems [169,170,171]. It is predicted that in 100 years, microplastic concentration in fertilized soil will reach 1159 mg kg^–1^ [172].

The adsorption and internal uptake of plastic particles have been confirmed in many plants used for human consumption, such as onion, wheat, and rice [173], posing a risk via trophic transfer [174]. There are several entry modes for microplastics into plants, notably through root adsorption, stomatal entry, and cracks or wounds [175].

Exposure of plants to microplastics can adversely affect many plant properties. It can impair germination [176], alter phytohormones concentration [177,178], reduce photosynthetic efficiency and biomass accumulation [179,180], affect fresh and dry weights of leaves and roots and change leaf number [181,182], prevent nutrient uptake by roots through lignification and cell atrophy [183], and cause ROS accumulation in tissues [184,185].

In wheat, MT treatment has been reported to effectively limit nanoplastics uptake and their systemic translocation by regulating aquaporin-related genes, specifically *TIP2-9*, *PIP2*, *PIP3*, *PIP1.2* in leaves and *TIP2-9*, *PIP1-5*, *PIP2*, and *PIP1.2* in roots. Through this mechanism, MT limits the transfer of nanoplastics through the transpiration stream. Furthermore, MT enhances carbohydrate metabolism and the ROS scavenging network by increasing the activities of APX and SOD in roots, which corresponded with lower H_2_O_2_ concentration, therefore neutralizing the growth-inhibitory effects of nanoplastic toxicity [186].

The protective role of melatonin has also been shown in recent studies regarding simultaneous microplastic and Cd exposure in maize and rice [187,188], as microplastic and heavy metal pollution usually coincides in urban-industrial regions. Microplastic contamination significantly promoted Cd uptake, causing degradation of the photosynthetic apparatus, oxidative instability, and suppressed growth and yield, whereas exogenous melatonin application decreased these negative effects, improved plant nutrient balance and chlorophyll synthesis, and supported antioxidant and hormonal balance, ultimately leading to better plant growth and yield.

The promising results of the existing research make it vital to continue pursuing this topic in order to fully understand plant resilience to micro- and nanoplastic toxicity. Based on the physiological changes analogous to those observed in previously described types of stress (particularly heavy metals), it can be theorized that under microplastic stress, MT application activates the same downstream defense systems. The involvement of NO is likely, considering the modifications in redox homeostasis. Consequently, exploring how melatonin application modulates endogenous NO fluxes under microplastic stress remains a highly promising direction for sustainable crop protection.

### 4.2. Nanoparticles

Nanoparticles (NPs) are materials measuring between 1 and 100 nanometers [189]. The functional properties of NPs depend on their size, shape, and structure [190]. Their properties can be optimized for various applications. These include biomedicine, agriculture, food technology, electronics, and defense industries [191]. NPs can be categorized into inorganic (metal and metal oxide NPs) or organic (polymeric or carbon-based NPs) types.

Nanotoxicology is a novel field of toxicology that focuses on the effects of nanosized particles on biological systems [192]. While the accelerated production of NPs offers advances in academic and industrial sectors, it simultaneously introduces complex ecological and public health challenges. Interestingly, some nanoparticles can mitigate the effects of other dangerous particles; for example, they can degrade microplastics [193]. Nanotechnology requires a dual-focus approach: maximizing industrial utility while minimizing the toxicological footprint on biological systems and society [194]. Although artificially designed NPs are supposed to be beneficial to plants, their entry into the cell is inseparably connected with some level of oxidative stress, as NPs permeate a cell, resulting in lipid peroxidation [195,196]. NPs interact with mitochondria, catalyze redox reactions, and dissolve, which in the case of metal-based NPs releases toxic ions, all of which increase ROS generation. Signaling in NP stress is also likely to depend on ROS emitted by the NPs, which trigger cytoplasmic Ca^2+^ ion channels, Ca^2+^/Na^+^–ATPases, and MAPK activation [197,198]. At low concentrations, NPs can alleviate heavy metal stress by altering root-to-shoot translocation and immobilizing metals in the rhizosphere. NPs can also bind heavy metals within root tissues and reduce their translocation to shoots. However, these interactions are strictly dose-dependent, with low NP concentrations supporting plant growth and metal detoxification, whereas high concentrations disrupt the cellular redox balance and metal chelation [199,200,201].

There is also a clear link between NPs-related stress and NO, as numerous reports have documented the protective effects of exogenous chemical NO donors (mainly SNP) on nanoparticle-induced stress in plants. Activation of the antioxidant system and promotion of plant development and growth have been reported [202,203,204]. Conversely, NPs can activate NR, resulting in higher endogenous NO levels [205]. NPs have also been shown to increase the activities of antioxidant enzymes, such as POX, CAT, and SOD [206], highlighting their role in altering the redox state of cells.

Furthermore, plant sensitivity to NPs has been shown to be species-specific, with nanoparticle perception being more effective in dicotyledons than in monocotyledons, depending on the cell wall architecture and stomatal arrangement [164,207]. Nanoparticles can also suppress seed germination, disrupt the photosynthetic apparatus, affect plant reproductive productivity and phytohormone accumulation, impair nutrient uptake, and mediate cyto-genotoxicity.

Although the toxic effects of NPs are well known, there is little research on possible ways to alleviate NP-related stress. The main protective strategy considered so far has been the use of NO donors. However, considering melatonin–NO interactions, exogenous melatonin has also been investigated as a protective agent against NP stress. Improved photosynthesis has been reported in wheat under ZnO NP stress, along with enhanced antioxidant enzyme activities and improved dry weight, total root length, and larger leaf area [207]. The same trend was observed in maize under CuO NP stress [208]. In rice under ZnO and CuO NP stress, exogenous MT increased the antioxidant enzyme activity and transcript levels of genes encoding these enzymes. Furthermore, the impairment of proline and arginine biosynthesis under NP stress was alleviated by melatonin treatment [209]. These studies provide promising insights into the potential of MT in alleviating more complex and emerging environmental stresses.

### 4.3. Greywater and Surfactants

In the face of increasing water scarcity worldwide, the search for alternative irrigation sources persists. The use of “greywater” appears to be a promising area of research, for both economic and environmental reasons. The term “greywater” refers to wastewater from all domestic sources, such as showers, washing machines, and kitchen sinks, except for toilet wastewater [210]. After proper treatment, greywater can be recycled, as long as it meets local water quality standards. Possible reuse options include toilet flushing, car washing, or laundry [211].

In recent years, the possibility of applying greywater for irrigation in agriculture has been a promising direction. The safety of greywater for ornamental plant irrigation has been demonstrated, with little to no negative effects on plant biomass or flowering [212,213], although leaf chlorosis and reduced chlorophyll content were reported.

Water quality is crucial, as untreated greywater can inhibit germination, unlike freshwater or treated greywater [214]. In addition to the public health risks, we must not overlook the potential environmental impacts of greywater use, including increased soil salinity stress [215] and heavy metal stress [216], as discussed in the previous chapter. There have been studies on the effects of greywater irrigation on tomatoes, both with untreated and treated greywater [217,218,219]. Treated greywater shows promising results, enhancing chlorophyll content, plant height, yield, and proline levels in plants, compared to untreated greywater. Applying greywater to *Propolis* species also resulted in higher fresh and dry weight in shoots compared to diluted greywater and control [220].

In hydroponics, cultivation of lettuce with nitrified urine and greywater resulted in growth and yield comparable to plants grown in standard nutrient solution [221]. Similarly, greywater co-treated with urine in an aerobic-activated sludge reactor and then diluted and supplemented with nutrients could be successfully used as a hydroponic medium for cultivating cucumbers, as it did not drastically affect plant growth [222]. Moreover, increased activity of NR, the main producer of NO, was observed, along with increased H_2_O_2_ levels, lipid peroxidation, and APX activity, indicating possible involvement of NO in facilitating plant response to greywater.

Surfactants are important components of greywater. They are detergents that reduce the surface tension between two phases (e.g., liquid and solid) by increasing the distance between the water molecules [223,224]. Surfactants have a wide range of applications in the chemical industry (as additives in cosmetics and detergents) and agriculture, where they are added to pesticides to facilitate dispersion [225]. The presence of surfactants in greywater, even after initial purification, poses a serious problem for its use, primarily due to their high concentrations, uncertain biodegradability, and impact on human health [226].

The effects of surfactants on plant growth have been studied in both soil-based and soilless cultivation. However, treatment outcomes depend on the type of surfactant used and its concentration. For example, using 5 g·L^−1^ of commercial laundry detergent, containing mostly linear alkylbenzene sulfonate (LAS), resulted in failure of cultivation after 12 days of planting for lettuce and after 20 days for okra, but using a low concentration (0.1 g·L^−1^) had only a moderate or limited impact on these plants [227]. Similarly, high concentrations (>1.0 g·L^−1^) of the surfactants SLES, SDBS, or SMCT can lead to plant death or severely reduced yield in lettuce, while low concentrations of the same surfactants (ca. 0.07 g·L^−1^) resulted only in moderately worsened plant yield (SLES, SMCT) or were harmless (SDBS) [221,228]. Based on preliminary research, SMCT-induced stress leads to an overproduction of NO in roots, which correlates with restricted plant growth and dehydration of tissues [229], highlighting the possible role of NO signaling in phytotoxic response to stress caused by surfactants.

It is worth mentioning that due to its specificity, greywater also contains microplastics [230,231], which further increase its risk factor, alongside the salt and heavy metal stress caused by greywater, as mentioned previously. However, given the protective role of melatonin against salinity and heavy metal stress, it has emerged as a viable candidate for improving the performance of greywater-irrigated crops. Implementing MT-based strategies could significantly alleviate the harmful effects of greywater and/or surfactants on plant growth and development.

## 5. Future Perspectives

While the individual roles of MT and NO are broadly documented, critical questions still remain regarding the precise architecture of their signaling pathways and the molecular mechanisms of their crosstalk. High-throughput genomic, transcriptomic, and proteomic analyses are required to resolve long-standing issues. At the genomic level, future efforts should focus on characterizing the promoter features of MT-responsive genes and identifying the specific regulatory elements of MT and NO biosynthetic enzymes. At the proteomic level, key priorities include identifying the covalent modification targets of both molecules (e.g., *S*-nitrosylation cascades) and expanding the identification of candidate MT receptors in different plants beyond the *Arabidopsis* CAND2/PMTR1 model. Resolving these molecular architectures will also help clarify the potential of exogenous NOMela application to substitute for the separate use of individual MT and NO donors.

Substantial progress has been made in understanding the physiological roles of melatonin and NO under “classical” abiotic stresses, including drought, salinity, low temperature, and heavy metal exposure. The results of these studies can be used to design advanced experiments that could help fight modern world environmental concerns. The ever-expanding range of anthropogenic pollutants, including microplastics, nanoparticles, surfactants, but also pharmaceuticals, electronic waste (E-waste), or per- and polyfluoroalkyl substances (PFAS), highlights the need for further investigation. Utilizing melatonin-based strategies represents a highly promising direction for improvement of plant resilience, crop productivity, and food safety in increasingly contaminated environments.

## Data Availability

No new data were created or analyzed in this study. Data sharing is not applicable to this article.

## Abbreviations

The following abbreviations are used in this manuscript:

MT	Melatonin
NO	Nitric oxide
RNS	Reactive nitrogen species
ROS	Reactive oxygen species
